# Projected cervical Cancer incidence in Swaziland using three methods and local survey estimates

**DOI:** 10.1186/s12885-018-4540-1

**Published:** 2018-06-07

**Authors:** Themba G. Ginindza, Benn Sartorius

**Affiliations:** Discipline of Public Health, School of Nursing and Public Health, University of KwaZulu-Natal, Howard College Campus, 2nd Floor George Campbell Building, Mazisi Kunene Road, Durban, 4041 South Africa

**Keywords:** Cervical cancer incidence, High risk human papillomavirus prevalence modelling, Swaziland

## Abstract

**Background:**

The scarcity of country data (e.g. a cancer registry) for the burden of cervical cancer (CC) in low-income countries (LCIs) such as Swaziland remains a huge challenge. Such data are critical to inform local decision-making regarding resource allocation [1]. We aimed to estimate likely cervical cancer incidence in Swaziland using three different methodologies (triangulation), to help better inform local policy guidance regarding likely higher “true” burden and increased resource allocation required for treatment, cervical cancer screening and HPV vaccine implementation.

**Methods:**

Three methods were applied to estimate CC incidence, namely: 1) application of age-specific CC incidence rates for Southern African region from GLOBOCAN 2012 extrapolated to the 2014 Swaziland female population; 2) a linear regression based model with transformed age-standardised CC incidence against hr-HPV (with and without HIV as a covariate) prevalence among women with normal cervical cytology; and 3) a mathematical model, using a natural history approach based on parameter estimates from various available literature and local survey estimates. We then triangulated estimates and uncertainty from the three models to estimate the most likely CC incidence rate for Swaziland in 2015.

**Results:**

The projected incidence estimates for models 1–3 were 69.4 (95% CI: 66.7–72.1), 62.6 per 100,000 (95%CI: 53.7–71.8) and 44.6 per 100,000 (41.5 to 52.1) respectively. Model 2 with HIV prevalence as covariate estimated a higher CC incidence rate estimate of 101.1 per 100,000 (95%CI: 90.3–112.2). The triangulated (‘averaged’) age-standardized CC incidence based across the 3 models for 2015 was estimated at 69.4 per 100,000 (95% CI: 63.0–77.1) in Swaziland.

**Conclusion:**

It is widely accepted that cancer incidence (and in this case CC) is underestimated in settings with poor and lacking registry data. Our findings suggest that the projected burden of CC is higher than that suggested from other sources. Local health policy decisions and decision-makers need to re-assess resource allocation to prevent and treat CC effectively, which is likely to persist given the very high burden of hr-HPV within the country.

**Electronic supplementary material:**

The online version of this article (10.1186/s12885-018-4540-1) contains supplementary material, which is available to authorized users.

## Background

The successful implementation of cervical cancer (CC) screening and the introduction of human papillomavirus (HPV) vaccine as a preventative strategy to reduce cervical cancer burden has had a great impact especially in the high-income countries (HIC) [1]. However, CC is still estimated to be the fourth most common cancer worldwide among women, with an increasing number of new cases: from 493,000 new cases in 2002 to 530,000 in 2008, and the number of deaths increasing from 274,000 in 2002 to 275,000 deaths in 2008 [2–4]. About 85% of the world’s CC cases occur in the low-income countries (LICs) [5]. CC is the most frequent cancer type among women in Africa, and highly prevalent among women ages 15 to 44 [6] and in the most disadvantaged population [7]. Over 90% of all cervical cancer cases are caused by persistent infection with high-risk types Human Papillomaviruses (HPV) [1, 6], which can lead to pre-cancerous lesions that may progress to invasive cervical carcinoma if left untreated [6].

The lack of data and poor quality data on CC are likely to result in an underestimated number of CC cases, since women often die of other competing causes, e.g. other AIDS defining illnesses, prior to cervical cancer diagnosis, and since the poor health infrastructure in many LICs results in under-reporting of CC [6]. However, quantifying the CC rate is a critical first step towards prevention as it provides vital information to policy and decision-makers when ascertaining all resources needed to tackle the disease [7, 8]. It has been established that the most accurate measure of CC incidence can be attained from population-based registries, which provide estimates of disease occurrence in a well-defined population [9]. Research has demonstrated that the quality and completeness of data collection, as well as accurate and reliable measures of population denominators are very crucial components for cancer registries [7]. Unfortunately, for LICs like Swaziland, the lack of proper resources and infrastructure for case findings and reporting prevent the establishment and maintenance of accurate cancer registries. Furthermore, such challenges in LICs have contributed to the fact that many cases of CC go undiagnosed and unreported [1, 2]. About 80% of cervical cancer patients in developing countries like Swaziland present with late-stage tumors when they are diagnosed, resulting in poor prognosis [2]. As means of cervical cancer screening, Pap smear was introduced in national cervical cancer prevention programme in 1983 [10]. However, in 2009, the government of Swaziland incorporated the “See and Treat” approach to quicken the early detection of cervical lesions and facilitate the extension of cervical cancer prevention services across four political regions [11].Currently, HPV vaccine is not part of Extended Programme on Immunization (EPI) in the country.

The understanding of the epidemiology and natural history of cervical cancer at population level and to prevent the escalating burden of the disease in LICs is essential. Scarcity of country data on the burden of cervical cancer remains a huge challenge in some LICs such as Swaziland, yet such data are critical to informing decisions about resource allocation to combat the disease. The lack of cancer registries to provide these data in LICs is the major limitation to establish cancer incidence.

The aim of this study is to develop a prediction model to estimate cervical cancer incidence without a population-based cancer registry, but using currently country detected hr-HPV prevalence and other continental prevalence. Measuring the CC burden is of paramount importance to better inform policy guidance on cervical cancer screening, as well as developing strategies on HPV vaccine implementation.

## Methods

### Estimation methods

In our study, we applied 3 methods to estimates the cervical cancer data:

For ***method 1***, we employed indirect standardization to estimate expected incidence in Swaziland by applying age-specific CC incidence rates for the Southern African region from GLOBOCAN 2012 estimates [12] to the 2014 Swazi female population structure [13] to obtain the expected number cases per age-group and to estimate CC incidence among women aged 30 + .These summed expected cases were scaled by the population total and multiplied by 100,000. ***Method 2***: an ecological regression model (e.g. [14]) was employed to regress age standardized CC incidence at country level from GLOBOCAN 2012 [12] in sub-Saharan Africa (SSA) countries against hr-HPV prevalence among women with normal cervical cytology [15] and including additional covariates such as HIV prevalence and adolescent birth rate. ***Method 3***: a mathematical natural history model based on 3 scenarios (average, best and worst case) as part of the sensitivity analysis. Further details are provided below under the statistical methods section as well as in Additional file 1.

### Data collection

Different countries’ age-specific prevalence data on HPV infection were available. We obtained the following data:

#### HPV prevalence

HPV prevalence estimates for Swaziland were obtained from a local survey undertaken between June and July, 2015. The main aim of this survey was to estimate prevalence and identify associated determinants of hr-HPV, including HIV infection [16]. A total of 655 women aged between 15 and 49 years from five health facilities were randomly enrolled using a cross-sectional study design. Cervical cells were tested for hr-HPV types using GeneXpert HPV Assays. Age and region-weighted analyses were done to estimate the overall hr-HPV prevalence and co-infection with HIV infection given the stratified systematic random sampling design. Survey weighted analysis was done to adjust the sample characteristic to match up with the population (age 15–49 years) that they were selected to represent. Other prevalence of HPV infection was derived from a meta-analysis of age-specific HPV prevalence in 1 million women with normal cytology; methods are detailed elsewhere [1]. Prevalence of hr-HPV among women with normal cervical cytology in Africa by country was also utilised [15].

#### Cervical cancer incidence

Since no local cancer registry data (especially age-standardized CC incidence (ASR)) were available for Swaziland, we extracted age-specific cervical cancer incidence rates for available countries from GLOBOCAN 2012 [12] for both use in methods 1 (indirect standardization approach) and for method 2 (regression against prevalence of hr-HPV among women with normal cytology).

### Statistical analysis

Statistical analyses were done using Stata 13.0SE (Stata Corp.College station, Texas, USA). To summarize the strength of the linear correlation between country’s hr-HPV in women ages 15–49 and CC incidence rates we used the Spearman rank correlation coefficient (r). Furthermore, an ecological country level linear regression model was used to predict cervical cancer incidence from hr-HPV prevalence. The hr-HPV prevalence estimate from the aforementioned survey (namely 46.2%) was then used to on the fitted line to estimate the age-standardized incidence in Swaziland. The dependent variable was checked for normality and best transformation (square root) applied. A model with local hr-HPV prevalence and HPV prevalence among women with normal cytology from 5 continents, predicting CC incidence was considered the “base model”. In our analysis we further restricted the regression analysis between age standard incidences of cervical cancer (Swazi ASR estimate from GLOBOCAN-2012) [12] vs HPV prevalence among women with normal cytology from African countries [1] given the relatively higher burden in Africa and the potential for underestimation if more developed settings are included. In addition, we also run a version of this model with HIV prevalence as covariate to account for the potential population level impact attributed to enhanced HPV carcinogenesis due to HIV-related immunosuppression.

The mathematical model for the natural history of HPV infection and cervical carcinogenesis (decision tree framework) was implemented in Tree Age Pro using a Markov modeling approach [17, 18]. A Markov process is characterized by specifying the finite set of possible states and the stationary probabilities of transition between these states (progression and regression) as well as retention in the current state. We employed a decision tree approach which was composed of 7 health states [19], reflecting the natural history of the disease: no infection (healthy), infection with an oncogenic HPV virus without precancerous or cancerous lesion; cervical intraepithelial neoplasia (CIN) grade 1; CIN grade 2 or 3; persistent CIN grade 2 or 3; CC; diagnosed CIN grade 1 through screening; diagnosed CIN grade 2 or 3 through screening; diagnosed persistent CIN grade 2 or 3 through screening; CC; death from CC. A diagrammatic representation of the model structure used in presented in Additional file 1. The states and natural history transition probabilities employed are shown in Table 2. We also developed a table with various annual progression and regression probabilities based on previous studies and available literature. As part of our sensitivity analysis we used both the mean value for each parameter based on available literature and context specific prevalence estimates as well as the min and maximum parameter values identified (either in the literature or based on the 95% CI off the survey parameter used e.g. hr-HPV prevalence in Swaziland based on the aforementioned survey that was conducted by the lead author. These yielded the 3 different scenarios alluded to earlier, namely: most likely, best and worst case.

## Results

### Model 1: Age-specific CC incidence rate for the southern African region extrapolated to the 2014 Swaziland female population

**Fig. 1:**
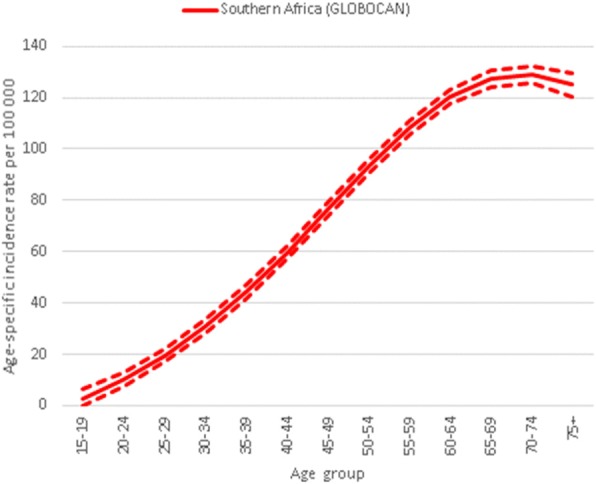
Age-specific cervical cancer incidence rate for the Southern African region

The age-specific incidence rates by age group for Southern Africa from GLOBOCAN 2012 are presented in Fig. 1. The overall annual expected number of incident CC cases in Swaziland was 106 (95%CI: 101–110) and the CC incidence rate was estimated at 68.5 per 100,000 (95%CI: 65.7–71.2) among women age 30+ (Table 1).

**Table 1:** Expected number of cervical cancer estimates of women in Swaziland extrapolated to Swazi female population based on 2014 structure

Age group	Pop (2014)^a^	Age specific incidence rate for Southern African^b^	Expected number of cases	Lower	Upper
30–34	46,793	30.96031	14.48726	13.25555	15.71896
35–39	37,472	44.4069	16.64015	15.65899	17.62132
40–44	29,484	59.72654	17.60977	16.88616	18.33339
45–49	22,960	76.32104	17.52331	16.93195	18.11467
50–54	17,655	93.06319	16.43031	15.9327	16.92791
55–59	13,765	108.3291	14.9115	14.53422	15.28878
60–64	10,523	120.2819	12.65726	12.36527	12.94926
65–69	7935	127.3817	10.10774	9.853357	10.36212
70–74	5592	128.8913	7.207601	7.035971	7.379232
75+	7081	125.0797	8.856894	8.531789	9.181999
Overall (30+)	199,260		136.4318	130.986	141.8776
Incidence (30+) per 100,000	68.5 (95% CI: 65.7–71.2)				

^a^Extrapolated to the 2014 Swaziland female population structure from the Swaziland Population Projections 2007–2030

^b^Estimates from GLOBOCAN 2012 report

### Model 2: Linear regression model

**Fig. 2:**
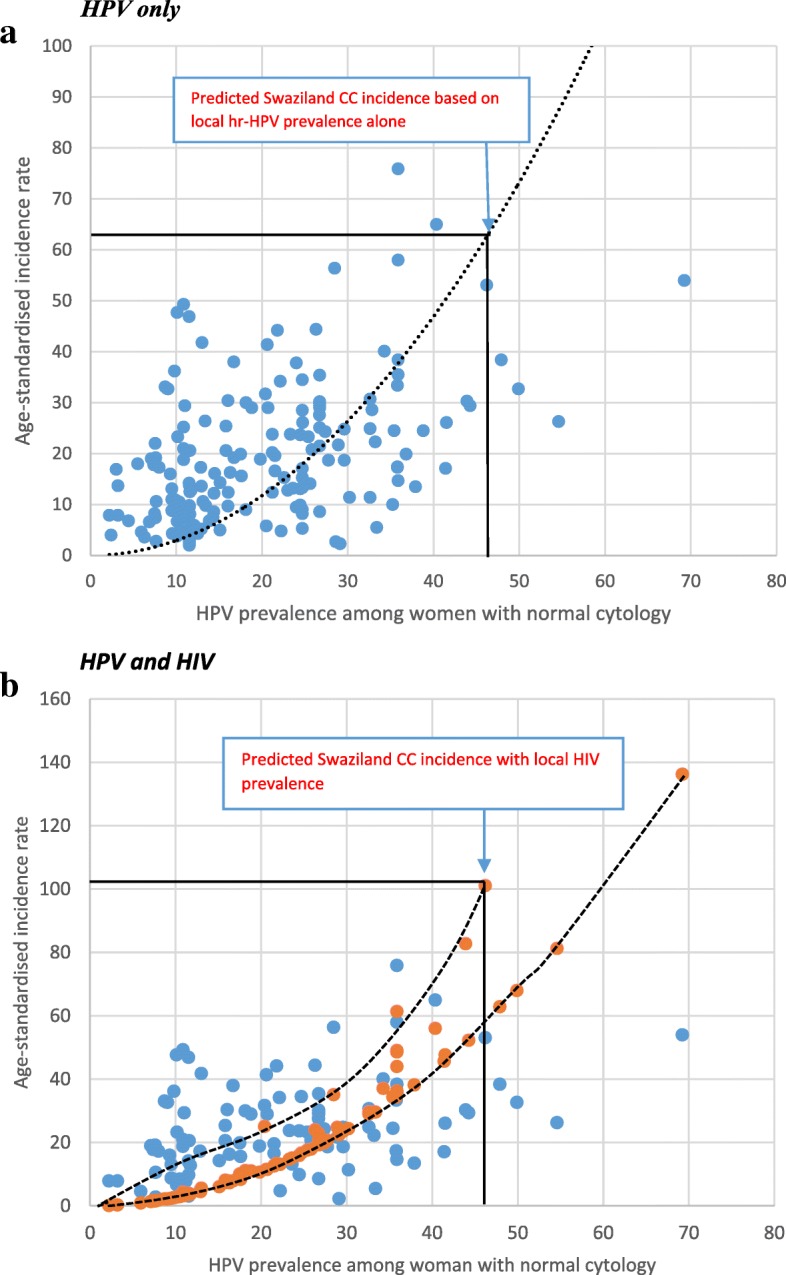
Showing the association between HPV prevalence among women with normal cytology from African countries and standardized CC incidence in women ages 15–75+. HPV only. HPV and HIV

The ASIR was not normally distributed; however, a square root transform corrected this issue. We thus used square rooted ASIR as the dependent variable in the model. Figure 2 highlights the strong relationship between hr-HPV prevalence among women with normal cytology and age standardised cervical cancer incidence among African counties with available data. We observed a moderate positive correlation between ASIR and HPV (Spearman rank correlation coefficient [r] = + 0.44, *p* < 0.001). The model (without HIV as a covariate) estimated an age-standardized cervical cancer incidence of 62.6 per 100,000 women (95%CI: 53.7–71.8) in Swaziland (Fig. 2a). In the model which included HIV as a covariate the projected age standard incidence increased to 101.1 per 100,000 (95%CI: 90.3–112.2) (Fig. 2b).

### Model 3: Mathematical natural history Markov model

The parameter values used in the three different scenarios are presented in Table 2: scenario 1, the scenario containing the annual average progression/regression of all natural history parameters; scenario 2, using the minimum value for all natural history parameters; and scenario 3; the worst case scenario where the maximum values for all natural history parameters were utilized. Based on scenario 1, 2 and 3, the estimated age-standardized cervical cancer incidence was estimated at 44.6 per 100,000; 41.5 per 100,000 and 52.1 per 100,000 respectively.

**Table 2:** Model of Natural History Parameters: Annual Average

Parameters calibration	Average	Min	Max	Source (Reference no.)
Baseline calibration				
Well to hr-HPV	46.2%	42.8%	49.5%	[16]
HPV16 and/or 18	25.9%	20.0%	33.4%	
CIN1	4.4%	3.0%	5.5	
CIN2	0.6%	0.1%	2.9%	
CIN3	0.6%	0.12%	2.9%	
invasive cervical cancer	0.5	0.5%	0.5%	
Progression from well to..				
hr-HPV infection	6.1%	0.0%	14.0%	[20, 21]
Progression from hr-HPV (12 types) to.				
to CIN1	6.3%	5.0%	7.9%	[20–22]
to CIN2	0.1%	0.1%	0.1%	[23]
to CIN3	1.1%	0.1%	2.0%	[23, 24]
Progression from hr-HPV 16/18 to.				
to CIN1	9.9%	9.9%	9.9%	[25, 26]
to CIN2	0.6%	0.6%	0.6%	[23]
to CIN3	1.5%	1.5%	1.5%	[23]
Progression from CIN1				
to CIN2	5.2%	1.0%	13.6%	[21, 22, 26–30]
to CIN3	10.1%	0.9%	29.0%	[21, 22, 27, 28, 30–33]
Progression from CIN2				
to CIN3	9.1%	4.2%	14.0%	[26–29, 34, 35]
to ICC	3.4%	0.2%	10.0%	[21, 22, 27, 28, 34–36]
CIN3 to Invasive Cervical Cancer	2.6%	1.1%	4.1%	[26–28, 37]
Annual mortality rate for cervical cancer^a^	6.4%	3.1%	60.1%	[18, 38]
Regression from hr-HPV (12 types) to.				
with normal smear to well	50.3%	42.0%	58.6%	[21, 39]
with mild smear to well	45.6%	45.6%	45.6%	[39]
Regression from hr-HPV to.				
with normal smear to well	37.7%	31.6%	43.8%	[24, 39]
with mild smear to well	21.8%	21.8%	21.8%	[39]
Regression from CIN1				
to well	42.9%	9.8%	78.0%	[21, 22, 26, 28, 30, 31, 33, 36, 39]
to hr-HPV	4.9%	2.4%	7.3%	[27, 28, 36, 40]
Regression from CIN2				
to well	20.4%	9.4%	38.0%	[21, 22, 24, 26–28, 35, 36, 41, 42]
to CIN1	11.4%	9.4%	13.3%	[26–28, 35, 36, 41, 42]
Regression from CIN3				
to well	3.9%	3.9%	3.9%	[27, 37]
to CIN1	2.3%	1.6%	3.0%	[26, 27, 37]
to CIN2	3.0%	3.0%	3.0%	[26]

Hr-HPV: high risk human papillomavirus; CIN: cervical intraepithelial neoplasia; ICC: Invasive Cervical Cancer

^a^Average range of annual mortality rate for cervical cancer

### Final estimated age-standardized CC incidence rate

Table 3 showing cervical cancer incidence estimates from all the three models. The indirect standardization and ecological regression based approach (without HIV as a covariate) yielded fairly similarly age standardized incidence estimate of 68.5 and 62.5 respectively and this difference was not statistically significant given the overlap of the 95% CI’s. When HIV was included as a covariate in the ecological regression, approach the projected incidence based on method 2 increased significantly to 101 per 100,000. The natural history approach (method 3) yielded a significantly lower incidence estimate compared to methods 1 and 2. TA triangulation of the 3 approaches was employed to estimate the most likely CC incidence in Swaziland in 2014.The triangulated annual age-standardized CC incidence was estimated at 58.6 per 100,000 (95% CI: 53.6.0–65.0) in Swaziland when the ecological model estimates were included without HIV. If we translate this incidence rate to the Swaziland female population aged 30+ for 2015 this would likely have yielded 117 (95% CI: 107–130) incident cases in 2014 among women aged 30 + .

**Table 3:** Summary estimates of the models

Models	Estimates per 100,000	Lower bound	Upper bound
1	69.4	66.7	72.1
2a	62.6	53.7	71.8
2b	101.1	90.3	112.2
3	44.6	41.5	52.1
Triangulation 1:	58.9	54.0	65.3
Triangulation 2:	69.4	63.0	77.1
Number incident cases for female Swaziland population 30+ in 2014 (pop size 152,892)	106	96	118
Number incident cases for female Swaziland population 15+ in 2014 (pop size 318,819)	221	201	246

Model 2a: with HPV prevalence only; Model 2b: with HPV and HIV; Triangulation 1: 1+(2a+2b)+3: with HIV estimate i.e. 2a+b averaged prior to triangulation with models 1 and 3; Triangulation 2: 1+(2a+2b)+3 (with HIV estimate i.e. 2a+b averaged prior to triangulation with models 1 and 3)

## Discussion

Cervical cancer remains a significant public health concern worldwide especially in the low-income countries [43, 44]. Continental reports or studies on the incidence of cervical cancer have demonstrated the severity of the HPV related condition [45]. It has been established that population-based cancer registries are a source for quantifying the disease burden in a defined population. However, the most regrettable situation is that cancer registries are either non-existent or not fully operational in most LCIs such as Swaziland, thus preventing the estimation of the actual disease burden [43, 44]. Therefore, the use of available HPV prevalence and other HPV natural history parameters data to predict cervical cancer incidence become of paramount importance to cover such a gap. Hence, our study used the local and other African countries’ HPV prevalences to predict cervical cancer incidence for Swaziland. Our study demonstrated, as anticipated, a significant linear correlation between population prevalence of hr-HPV infection and cervical cancer incidence. Our study established that HPV among women with normal cytology is a strong predictor of cervical cancer incidence. Based on the three models triangulation approach employed in this study, the predicted average annual age-standardized CC incidence was 58.6 per 100 00 in Swaziland. However, after factoring current HIV prevalence into the model, a higher CC incidence rate estimate of 65.0 per 100000was estimated.

### Strengths of the study

This is the first study in Swaziland to estimate the incidence of cervical cancer utilizing local hr-HPV prevalence data and other African countries’ data. In addition, we used 3 accepted methods to triangulate a “best guess” estimate. Furthermore, we sourced multiple estimates for the natural history model to try getting the best-weighted estimates for the progression/regression parameter values and also performed a sensitivity type analysis. The further novelty of our study is that we factored HIV in the model to estimate the impact of HIV on the incidence rate of cervical cancer in Swaziland.

### Weakness of the study

The key limitations of our study was that our findings are likely to underestimate the incidence rate since our hr-HPV prevalence was obtained from women of the ages between 15 and 49, yet studies have shown that prevalence at later ages tend to show a better prediction of CC incidence. Another limitation of our study is the effect of ecological fallacy relating to model 2. Furthermore, the age specific CC incidence rates for CC may not be same as in Swaziland (very much biased towards South Africa). However, we have similar burdens for HIV/hr-HPV: most of the countries across the southern African region have experienced high HIV and HPV infection. Another limitation is that we did not factor in HIV. However, future work indicates that we will attempt to refine these estimates including HIV parameter/stratification in all modelling approaches. Finally, the mathematical model: the parameter values may be more biased to more developed settings and hence underestimate CC transition probability.

This current study found a strong correlation between the current population hr-HPV prevalence among women with normal cytology and age standardized cervical cancer incidence. These findings are analogous to those observed from the past epidemiological studies [7, 46]. However, Sharma et al. demonstrated the age factor in the HPV correlation, where HPV prevalence at later ages was found to be an excellent predictor of cervical cancer incidence compared to that of women below the age of 35 years, with prevalence in women age 55–64 presenting the strongest correlation [7]. Such high risk could be due to a longer persistence of hr-HPV among old age women. Scientific evidence has been presented that the persistence of hr-HPV acutely increases the risk of developing cervical cancer [7, 47–49].

Our study presented, as expected, a predicted high age-standardized cervical cancer incidence (69.4 per 100,000) among the population in Swaziland. Our results were slightly higher than the ASR estimates provided by the GOBOCAN 2012 (53.1 per 100,000) [50]. These discrepancies might be due to the fact that our study used actual data as compared to the use of standard population or the rates of from neighboring countries or registries in the same area. In addition, the GLOBOCAN data is not stratified by HIV. Comparing our findings with the GBD 2015 (58.1 per 100,000, 95%CI: 17.3–159.1) [51] our study triangulation estimate without HIV (58.6 per 100,000) were almost identical to GBD estimates. The further novelty of our study is after factoring current HIV prevalence in the model to estimate the impact of HIV on the incidence rate of cervical cancer in Swaziland, a huge increase of ASR CC incidence rate of 101.1 per 100,000 (95%CI: 90.3–112.2) was observed in the ecological model and could suggest that approaches that do not account for high co-infection of hr-HPV and HIV could potentially underestimate cervical cancer incidence in HIV hyper endemic settings, particularly in Southern Africa. The high ASR in the country may be due to the fact that the country is facing a high epidemic of HIV infection as well as an HIV link with high hr-HPV infection both of which are more likely to be persistent. Studies have established that due to the lack of access to relevant prevention approaches and the association with the HIV epidemic, cervical cancer incidence is expected to rise in the next two decades [52]. Women infected with HIV have an elevated risk of developing certain malignancies and those malignancies are found to be HPV-related, which reflects the high rate of co-infection with HPV in women with HIV [53].

When comparing our estimated number of incident cases for Swazi female population age 15+, our current study estimated 221 incident cases. Our estimates were in line with annual number of new cervical cancer (223) reported by the GLOBCAN 2012 [50, 54] and the average prevalent annual number of 220 reported by Swaziland National Cancer Registry in 2015 [55].

This current study reinforces the affirmation that a well conducted population-based HPV survey may possibly offer crucial information to estimate the risk of cervical cancer, more especially in the absence of or an inaccurate national registry data. Up-to-date and authentic cancer data are crucial to identify most the important considerations for cancer control strategies at the country level, therefore establishing a quality reporting system and legalizing cancer reporting at national level (in private and public health settings) and creating data linkage procedures with the newly established cancer registry will increase the coverage and quality registry in the country. Finally, the biggest implication of such high incidence is the large cost that will occur for public health care resources utilized for the management and treatment of cervical cancer in Swaziland. The higher the incidence of cervical cancer, the higher the economic burden of cervical cancer in the country.

## Conclusions

In conclusion, the observation of this study raises a concern over the burden of cervical cancer where reliable cervical cancer statistics are limited despite the current study showing the high prevalence of hr-HPV and HPV/HIV-coinfection among the Swazi reproductive age women. Our model provided an overall estimate of cervical cancer incidence that can be functional to inform health policy decisions and decision-makers on the allocation of limited resources to prevent and treat cervical cancer effectively. Finally, our study significantly showing the need for future research to modify the natural history model of cervical cancer to factor in HIV co-infection in hyper-endemic settings.

## Additional file

Additional file 1: Detailed description of methods 1–3. (DOCX 115 kb)
