# Supplementary material for: Projected cervical Cancer incidence in Swaziland using three methods and local survey estimates
Source: BMC Cancer. 2018 Jun 7;18:639. doi: 10.1186/s12885-018-4540-1 (PMC5992849; doi:10.1186/s12885-018-4540-1)
Supplement: Supplementary file 1 — Detailed description of methods 1–3. (DOCX 115 kb) [file 12885_2018_4540_MOESM1_ESM.docx]

**Additional file 1: Detailed description of methods 1-3**

**Method 1: Indirect standardisation approach using age specific Globocan cervical cancer incidence rates for Southern Africa.**

We employed **indirect standardization** to estimate expected incidence in Swaziland using age-specific CC incidence rates for the Southern African region from GLOBOCAN 2012 estimates [ Ferlay J SI, Ervik M, et al.: GLOBOCAN 2012: Estimated cancer Incidence, Mortality and Prevalence Worldwide 2012. In. Lyon, France: IARC,; 2012.] and applied to the 2014 Swazi female population structure [ The Kingdom of Swaziland Gorvernment and UNFPA: SWAZILAND POPULATION PROJECTIONS 2007-2030. In. Edited by Office CS; 2007.] to obtain the expected number cases per age-group and to estimate CC incidence among women aged 30+. We chose the Southern African region given the similarity in the underlying prevalence of HIV.

**The following incidence estimates for cancers among females in Southern Africa where extracted from Globocan 2012:**

The age specific incidence rates among females in the region where then applied to the relevant age structured in Swaziland in 2014 based on local census estimates. We first developed a fitted line by age (using a non-linear regression) to the observed incidence by age group in Southern Africa:

These smoothed estimates (plus 95% CI’s) where then applied to the observed female age structure in Swaziland in 2014. We estimated this incidence using the expected cell counts calculated above using indirect standardisation from Globocan incidence rates by age group for cervical cancer in Southern Africa. These expected incident case counts were summed for age groups 30+ and scaled by the corresponding population total of women in Swaziland in 2014 aged 30+ and multiplied by 100 000 to give this incidence.

Namely:

| **Age group** | **Pop (2014)** | **Fitted Incidence per 100 000 (Globocan 2012)** | **Expected number of cases**  **(population * incidence/100000)** | **L** | **U** |
| --- | --- | --- | --- | --- | --- |
| 30-34 | 46793 | 30.96031 | 14.48726 | 13.25555 | 15.71896 |
| 35-39 | 37472 | 44.4069 | 16.64015 | 15.65899 | 17.62132 |
| 40-44 | 29484 | 59.72654 | 17.60977 | 16.88616 | 18.33339 |
| 45-49 | 22960 | 76.32104 | 17.52331 | 16.93195 | 18.11467 |
| 50-54 | 17655 | 93.06319 | 16.43031 | 15.9327 | 16.92791 |
| 55-59 | 13765 | 108.3291 | 14.9115 | 14.53422 | 15.28878 |
| 60-64 | 10523 | 120.2819 | 12.65726 | 12.36527 | 12.94926 |
| 65-69 | 7935 | 127.3817 | 10.10774 | 9.853357 | 10.36212 |
| 70-74 | 5592 | 128.8913 | 7.207601 | 7.035971 | 7.379232 |
| 75+ | 7081 | 125.0797 | 8.856894 | 8.531789 | 9.181999 |
| Total | 199260 |  | 136.4318 | 130.986 | 141.8776 |
| Incidence per 100 000 (30+) |  |  | 68.46924 | 65.7362 | 71.20227 |

| **Age group** | **Pop (2014)** | **Incidence per 100 000 (Globocan)** | **Expected number of cases**  **(population * incidence/100000)** |
| --- | --- | --- | --- |
| 15-39 | 227206 | 11 | 24.99266 |
| 40-44 | 29484 | 55.2 | 16.27517 |
| 45-49 | 22960 | 75.7 | 17.38072 |
| 50-54 | 17655 | 94.5 | 16.68398 |
| 55-59 | 13765 | 110.1 | 15.15527 |
| 60-64 | 10523 | 120.8 | 12.71178 |
| 65-69 | 7935 | 126.5 | 10.03778 |
| 70-74 | 5592 | 127.2 | 7.113024 |
| 75+ | 7081 | 126.3 | 8.943303 |
| Overall | 342201 |  | 129.2937 |
| Incidence per 100 000 (15+) |  |  | 37.78296 |
| Incidence per 100 000 (40+) |  |  | 90.70048 |

**Method 2: Regression based modelling approach**

Given the causal link between hr-HPV and cervical cancer at an individual level we tried to model this effect at a population level (using an ecological regression approach) so that we could estimate age standardised cervical cancer incidence in countries like Swaziland which do not have available cancer registry data. We thus utilised the observed significant correlation between prevalence of high risk HPV and age standardised cervical cancer incidence in an ecological regression based modelling approach, employed previously for liver and lung cancer [Sartorius K, Sartorius B, Aldous C, Govender PS, Madiba TE. Global and country underestimation of hepatocellular carcinoma (HCC) in 2012 and its implications. Cancer epidemiology. 2015 Jun 1;39(3):284-90; Sartorius B, Sartorius K. How much incident lung cancer was missed globally in 2012? An ecological country-level study. Geospatial health. 2016 May 31;11(2).], to predict the likely age standardised incidence in Swaziland based on observed hr-HPV prevalence. Country level age standardised cervical cancer incidence at country level was the dependent variable in the model and we employed hr-HPV prevalence as the primary independent or explanatory variable. We further restricted the regression analysis to compare countries in Sub-Saharan African given the relatively higher burden in Africa and the potential for underestimation if more developed settings were also included the model. Furthermore given the known positive mediation effect of HIV on HPV infection and subsequent increased risk of cervical cancer we also tested HIV prevalence as a further covariate in the model to account for this effect and given the disproportionately higher prevalence of HIV in Southern Africa where Swaziland is located. Additionally earlier age of sexual debut is a known risk factor for HPV infection and we thus tested and incorporated country level adolescent birth rates as a proximal measure of earlier sexual debut at a population level. The latter covariate also significantly improved the overall fit of the regression model as based on R2 statistic.

We employed an ordinary linear regression model with age standardised cervical cancer incidence as the dependent variable hr-HPV and adolescent birth rate as explanatory covariates as well as another formulation with hr-HPV prevalence, adolescent and HIV prevalence. However as age standardised incidence was not normally distributed we had to transform it to normality. We employed the ladder of powers (Tukey, J. W. 1977. Exploratory Data Analysis. Reading, MA: Addison-Wesley) test to suggest which transformation would perform optimally. Based on this test a square root transformation was indicated. We checked regression assumptions using diagnostic tests for the linear regression formulation (e.g. Breusch-Pagan / Cook-Weisberg test for heteroscedasticity). Furthermore potential collinearity between covariates was assessed using the variance inflation factors. Furthermore to ensure the robustness of the predicted age standardised cervical cancer incidence rate in Swaziland we also implemented a Generalized Linear Latent And Mixed Model (GLLAMM) model formulation with the same covariates but with additional random effect country level to estimate the coefficients (and significance thereof) as well as to quantify/account for any unobserved heterogeneity in the data at country level.

**Method 3: natural history model using decision tree framework and Markov chain simulation**

Major advances in the biological and clinical understanding of cervical carcinogenesis have led to the development of new disease simulation models (e.g. natural history models) to inform policy decisions and cost-effectiveness strategies (Schiffman M. The need for forward-looking decision analyses to guide cervical cancer prevention. Cancer Epidemiol Biomarkers Prev. 2011;20(2):219–220). We implemented a mathematical model for the natural history of HPV infection and cervical carcinogenesis (decision tree framework) in Tree Age Pro using a Markov modeling approach (Debicki D, Ferko N, Demarteau N, Gallivan S, Bauch C, Anonychuk A, Mantovani L, Capri S, Chou CY, Standaert B et al: Comparison of detailed and succinct cohort modelling approaches in a multi-regional evaluation of cervical cancer vaccination. Vaccine 2008, 26 Suppl 5:F16-28.). A Markov process is characterized by specifying the finite set of possible states and the stationary probabilities of transition between these states (progression and regression) as well as retention in the current state. We employed a decision tree approach which was composed of 7 health states (Campos, N. G., Burger, E. A., Sy, S., Sharma, M., Schiffman, M., Rodriguez, A. C., … Kim, J. J. (2014). An Updated Natural History Model of Cervical Cancer: Derivation of Model Parameters. American Journal of Epidemiology, 180(5), 545–555. http://doi.org/10.1093/aje/kwu159), reflecting the natural history of the, namely: no infection (healthy), infection with an oncogenic HPV virus without precancerous or cancerous lesion; cervical intraepithelial neoplasia (CIN) grade 1; CIN grade 2 or 3; persistent CIN grade 2 or 3; CC; death from CC. A diagrammatic representation of the model structure used in presented below. The states and natural history transition probabilities employed are shown in Table 2 in the main text.
